# Gene expression profiling reveals a role of immune system and inflammation in innate and stress-induced anxiety-like behavior

**DOI:** 10.3389/fgene.2023.1173376

**Published:** 2023-05-16

**Authors:** Adrien Gigliotta, Kalevi Trontti, Juho Väänänen, Iiris Hovatta

**Affiliations:** SleepWell Research Program and Department of Psychology and Logopedics, Faculty of Medicine, University of Helsinki, Helsinki, Finland

**Keywords:** RNA-sequencing, anxiety-like behavior, immune system, inflammation, inbred mouse strain

## Abstract

Anxiety is an evolutionarily conserved response that is essential for survival. Pathological anxiety, however, is a maladaptive response to nonthreatening situations and greatly affects quality of life. The recent COVID-19 pandemic has increased the prevalence of anxiety symptoms and highlighted the urge to identify the molecular events that initiate pathological anxiety. To this aim, we investigated the extent of similarity of brain region-specific gene expression patterns associated with innate and stress-induced anxiety-like behavior. We compared the cortico-frontal (FCx) and hippocampal (Hpc) gene expression patterns of five inbred mouse strains with high or low levels of innate anxiety-like behavior with gene expression patterns of mice subjected to chronic social defeat stress. We found significantly large overlap of the Hpc but small overlap of the FCx gene expression patterns in innate and stress-induced anxiety, that however, converged onto common inflammation and immune system canonical pathways. Comparing the gene expression data with drug-gene interaction datasets revealed drug candidates, including medrysone, simvastatin, captopril, and sulpiride, that produced gene expression changes opposite to those observed in innate or stress-induced anxiety-like behavior. Together, our data provide a comprehensive overview of FCx and Hpc gene expression differences between innate and stress-induced anxiety and support the role of inflammation and immune system in anxiety-like behavior.

## 1 Introduction

Anxiety disorders include panic disorder, social anxiety disorder, specific phobias, agoraphobia, and generalized anxiety disorder. They are ranked amongst the top 25 leading causes of burden worldwide and one-third of the population is affected by an anxiety disorder during their lifetime (Bandelow and Michaelis, 2015; Vos et al., 2020). Critically, the recent COVID-19 pandemic has doubled the prevalence of clinically significant anxiety symptoms in children and adolescents (Racine et al., 2021). Anxiety disorders are treated with drugs and/or cognitive behavioral therapy, but efficient and long-lasting treatments are still lacking. Thus, the major challenges are to identify the molecular events that initiate and maintain pathological anxiety, and to determine how to normalize this pathology. Accordingly, there is a need to find novel, well-defined, and clinically relevant drug targets.

Anxiety is an evolutionarily conserved response and some aspects of it can therefore be measured in mice (Price, 2003; Campos et al., 2013). Whereas innate anxiety is essential for survival in threatening environments, pathological anxiety is a prolonged, maladaptive, and exacerbated response to non-threatening situations. In humans, innate or trait anxiety is a characteristic of an individual’s personality, and it influences the tendency to respond with concerns and worries to various situations. People reporting high trait anxiety have a higher risk of developing psychiatric disorders compared to individuals with low trait anxiety (Chambers et al., 2004; Wang et al., 2019). Predisposition to anxiety disorders is affected by both genetic and environmental factors, such as adverse family environment, lower socioeconomic status, acute severe trauma, and chronic psychosocial stress (Bouwknecht and Paylor, 2002; Lutz et al., 2017). Preclinical animal models allow the study of both genetic and environmental risk factors of anxiety disorders in controlled experiments. In addition, the ease of access to the brain tissue allows the investigation of the cellular and molecular mechanisms underlying anxiety.

The critical influence of the genetic background on innate anxiety-like behavior has been established in studies that investigated several inbred mouse strains (Bouwknecht and Paylor, 2002; Hovatta et al., 2005; Wahlsten et al., 2006; Milner and Crabbe, 2008). The extent of anxiety-like behavior can be assessed using behavioral tests that are based on the natural propensity of mice to avoid bright and open areas (the open field, light-dark box, and elevated plus/zero maze tests) or indulge in social interactions (the social interaction test) (Bailey and Crawley, 2009). Stress-induced anxiety-like behavior has been investigated using various paradigms, such as the chronic social defeat stress (CSDS) (Avgustinovich et al., 2005; Krishnan et al., 2007), chronic restraint stress (Hill et al., 2013), and chronic unpredictable mild stress (de Andrade et al., 2013). Innate and stress-induced anxiety are regulated at least partially by the same brain regions, including the frontal cortex (FCx) and hippocampus (Hpc) (Chattarji et al., 2015). However, it is unclear whether the same molecular mechanisms and biological processes underlie both types of anxiety. The identification of these mechanisms is crucial for the development of better therapeutic strategies to alleviate anxiety.

To determine the extent of similarity of brain gene expression patterns associated with innate and stress-induced anxiety-like behavior, we investigated FCx and Hpc gene expression patterns in two mouse models. As the model of innate anxiety, we used five inbred mouse strains with high (A/J, 129S1/SvImJ, and DBA/2J) or low (C57BL/6J, FVB/NJ) levels of innate anxiety-like behavior (Hovatta et al., 2005). As the model of stress-induced anxiety, we used C57BL/6NCrl and DBA/2NCrl mice subjected to CSDS (Laine et al., 2018). To identify biological pathways involved in innate and stress-induced anxiety, we carried out comprehensive bioinformatic analysis of RNA-sequencing data obtained from these models. We found distinct gene expression patterns in innate and stress-induced anxiety-like behavior, which however, converged onto common inflammation and immune pathways. To identify drugs that may ameliorate innate or stress-induced anxiety, we carried out gene-drug enrichment analysis. We discovered several drugs that induced opposite gene expression patterns to innate (medrysone and simvastatin) or stress-induced (captopril and sulpiride) anxiety-like behavior.

## 2 Materials and methods

### 2.1 Dataset 1: innate anxiety-like behavior

#### 2.1.1 Animals and behavior

Three male mice from A/J, 129S1/SvImJ, DBA/2J, C57BL/6J, and FVB/NJ inbred mouse strains each were ordered from the Jackson Laboratory. Mice were let to acclimatize for 1 week before behavioral testing or dissections. They were singly housed during this period to minimize gene expression changes due to social hierarchy. We have previously measured anxiety-like behavior of these strains by the open field and light-dark box tests (Hovatta et al., 2005). Based on these results, we considered A/J, 129S1/SvImJ, and DBA/2J mice anxious and C57BL/6J and FVB/NJ mice non-anxious. All animal care conformed to the European Communities Council Directive 86/609/EEC, and the experiment was approved by the Laboratory Animal Center of the University of Helsinki.

#### 2.1.2 Sample preparation

Seven-week-old naïve mice were killed by cervical dislocation between 8 a.m. and 11 a.m. FCx and Hpc were dissected on a chilled Petri dish. FCx included 2 mm of the anterior part of the cortex and the Hpc was dissected whole. Samples were frozen in liquid nitrogen and stored at −80°C. RNA was extracted with TriReagent (Invitrogen) according to the manufacturer’s instructions, followed by quality control (Bioanalyzer RNA 6000 Nano, Agilent).

#### 2.1.3 RNA-sequencing

The sequencing libraries were prepared with the Nextera RNA Library Preparation Kit (Illumina), and sequenced (paired-end, FCx 93 bp, Hpc 101 bp) on Illumina HiSeq 2000 (Illumina) by the Finnish Institute for Molecular Medicine (FIMM) Technology Centre. Sequence reads were aligned to genome GRCm38 with STAR aligner 2.5.2b (Dobin et al., 2013), counting reads per gene (option GeneCounts) using GTF version 85. Low abundant transcripts were filtered out to keep genes with >2 counts per million (CPM) in at least 4 samples. Using these criteria, we detected 14,757 genes in the FCx, and 15,021 in the Hpc.

#### 2.1.4 Differential gene expression analysis

Differential expression analyses on normalized gene expression values (voom + TMM) were performed using limma eBayes (Ritchie et al., 2015; Phipson et al., 2016). Samples (N = 9) from innately anxious strains (A/J, 129S1/SvImJ, and DBA/2J strains; n = 3/strain) were compared to those (N = 6) from non-anxious strains (C57BL/6J, FVB/NJ; n = 3/strain). Criterion for differential expression was adjusted *p*-value (Benjamini–Hochberg) < 0.05, unless specified otherwise.

### 2.2 Dataset 2: chronic psychosocial stress-induced anxiety

#### 2.2.1 Animals and behavior

We used our previously published RNA-seq data set from the medial prefrontal cortex and ventral hippocampus of C57BL/6NCrl and DBA/2NCrl male mice after CSDS (Laine et al., 2018) Briefly, in the CSDS, a test mouse is introduced into the cage of a resident mouse (from CD1 strain), which displays aggressive behavior towards the intruder for up to 10 min. The test mouse and the resident mouse are then separated by a plexiglass wall pierced with holes allowing sensory exposure for 24 h. This procedure is repeated for 10 consecutive days with a novel resident mouse each day. On day 11, social avoidance test is carried out to quantify the amount of social interaction with an unknown CD1 mouse. Defeated mice are placed in an open arena with an empty plexgilass cylinder pierced with holes. We compared the amount of time that the defeated mice spent in the interaction zone (semi-circle around the cylinder) when the cylinder was empty (first trial) to the time spent when an unknown CD1 mouse was present inside the cylinder (second trial). We calculated the social interaction ratio (SIR) as follow:

$$SIR=\frac{Time\;spent\;in\;interaction\;zone\;with\;empty\;cylinder}{Time\;spent\;in\;interaction\;zone\;with\;unknown\;mouse\;in\;the\;cylinder}\times 100$$

The mean and standard deviation for the SIR were obtained after subjecting control mice from each strain to the social interaction test (C57BL/NCrl N = 123 and DBA/2NCrl N = 114, (Laine et al., 2018). Mice with a SIR within 1 standard deviation from the mean of the control mice were classified as stress resilient (resembling controls) while mice with a SIR below 1 standard deviation from the same-strain controls’ mean were categorized as stress susceptible as they displayed social avoidance.

#### 2.2.2 Sample preparation

After the last CSDS session, mice were singly housed for 1 week and killed by cervical dislocation between 8 a.m. and 11 a.m. Their medial prefrontal cortices (5 mm of either side of the midline between bregma 1.94—1.40) and ventral hippocampi (from bregma −3.40 to −3.88) were dissected on a chilled Petri dish and flash frozen in liquid N2. RNA was extracted with TriReagent (Cat# 15596026, Invitrogen™) followed by RNA quality control (2,100 Bioanalyzer, Agilent Technologies) using Agilent RNA 600 Nano Chip kit (Agilent Technologies).

#### 2.2.3 RNA-sequencing

rRNA was depleted with Ribo-Zero Gold rRNA Removal kit (Illumina Inc.) and sequencing libraries were prepared with Nextera (Illumina; ventral hippocampus) or ScriptSeq v2 (Epicentre; medial prefrontal cortex) RNA-seq library preparation kits. Sequencing was conducted on HighSeq 2000 (ventral hippocampus, paired-end 91 bp, Illumina) or NextSeq 500 platforms (medial prefrontal cortex, single-end 96 bp; Illumina). Low abundant genes were filtered, keeping genes with at least 1 CPM in at least six samples. With this threshold, we detected 18,947 genes in the medial prefrontal cortex and 19,560 genes in the ventral hippocampus.

#### 2.2.4 Differential gene expression analysis

Differential expression analyses were performed on normalized gene expression values (TMM + voom) using limma eBayes. We compared the C57BL/6NCrl susceptible mice to corresponding controls and DBA/2NCrl susceptible mice to corresponding controls. Criterion for differential expression was set to nominal *p*-value < 0.05, unless specified otherwise.

### 2.3 Comparison of innate vs. stress-induced anxiety

Since the dissected brain regions of dataset 1 encompassed a larger area of the same structures dissected in dataset 2, we first visualized the expression levels of known marker genes for neurons and glial cell types, to verify that there were no significant overall differences between the datasets (Supplementary Figure S1).

#### 2.3.1 Rank-rank hypergeometric overlap analysis

We applied rank-rank hypergeometric overlap analysis [R package RRHO v1.34, (Tsankova et al., 2006),] to compare overall gene expression differences between innate and stress-induced anxiety. RRHO infers pair-wise similarity between two differential gene expression lists. After differential expression analysis between innately anxious and non-anxious (dataset 1) or stress-susceptible and control comparisons (dataset 2), we ranked genes by their (-log10(p)*logFC). Only genes expressed in both innate and induced data sets were included (FCx = 13,680 genes, Hpc = 14,165). Significance of overlapping genes within bins of 100 genes between two ranked differential expression lists was then calculated by RRHO.

#### 2.3.2 Pathway analysis

We used Ingenuity Pathway Analysis (IPA v. 70750971 November 2021 release; Qiagen, Hilden, Germany (Krämer et al., 2014) to determine the enrichment of differentially expressed genes within functional pathways. The 1,000 genes with the largest |logFC|*p-value score for each comparison were used as the input gene lists. Analyses were run with evidence based on mammals and restricted to “Neurons”, “Nervous system” and “CNS Cell lines” tissues. Additional analysis settings were inclusion of only experimentally observed pathways, and both direct and indirect interactions.

#### 2.3.3 Drug-gene set enrichment analysis

To detect drugs and compounds that produce similar or opposite gene expression pattern with our differentially expressed genes, we carried out drug-gene enrichment analysis using the DSigDB (Yoo et al., 2015). We downloaded the drug-gene set interactions using the webtool (http://dsigdb.tanlab.org/DSigDBv1.0/) and used them as custom sets for GSEA Pre-ranked module, implemented in GSEA Desktop v4.2.3 (Subramanian et al., 2005). The pre-ranked GSEA was performed with 1,000 permutations, and we considered drug-gene sets with at least 3 differentially expressed genes in the innate or stress-induced differential expression gene lists and with familywise-error rate (FWER) < 0.05 as significant. FWER is the most conservative *p*-value obtained after GSEA reflecting the probability of making type I error (Benjamini and Hochberg, 1995).

#### 2.3.4 Drug and chemical compound discovery

To identify drugs and chemicals that induce gene expression differences similar (or opposite) to those detected in our experiments, we used the webtool CLUE Query (https://clue.io/query), a reference perturbagen signature database, based on the Connectivity Map database (CMap) (Subramanian et al., 2017). Gene input lists consisting of the 150 most up- or downregulated [ranked by -log10 (*p*-value)*logFC)] genes for each comparison were used as input (maximum number of genes for the tool). Only drugs and compounds with a negative raw connectivity score and with known mechanism of action were considered. We searched for their therapeutic use from the DrugBank (Wishart et al., 2018) and PubChem (Kim et al., 2021) databases.

## 3 Results

### 3.1 Mouse model of innate anxiety-like behavior

**FIGURE 1:**
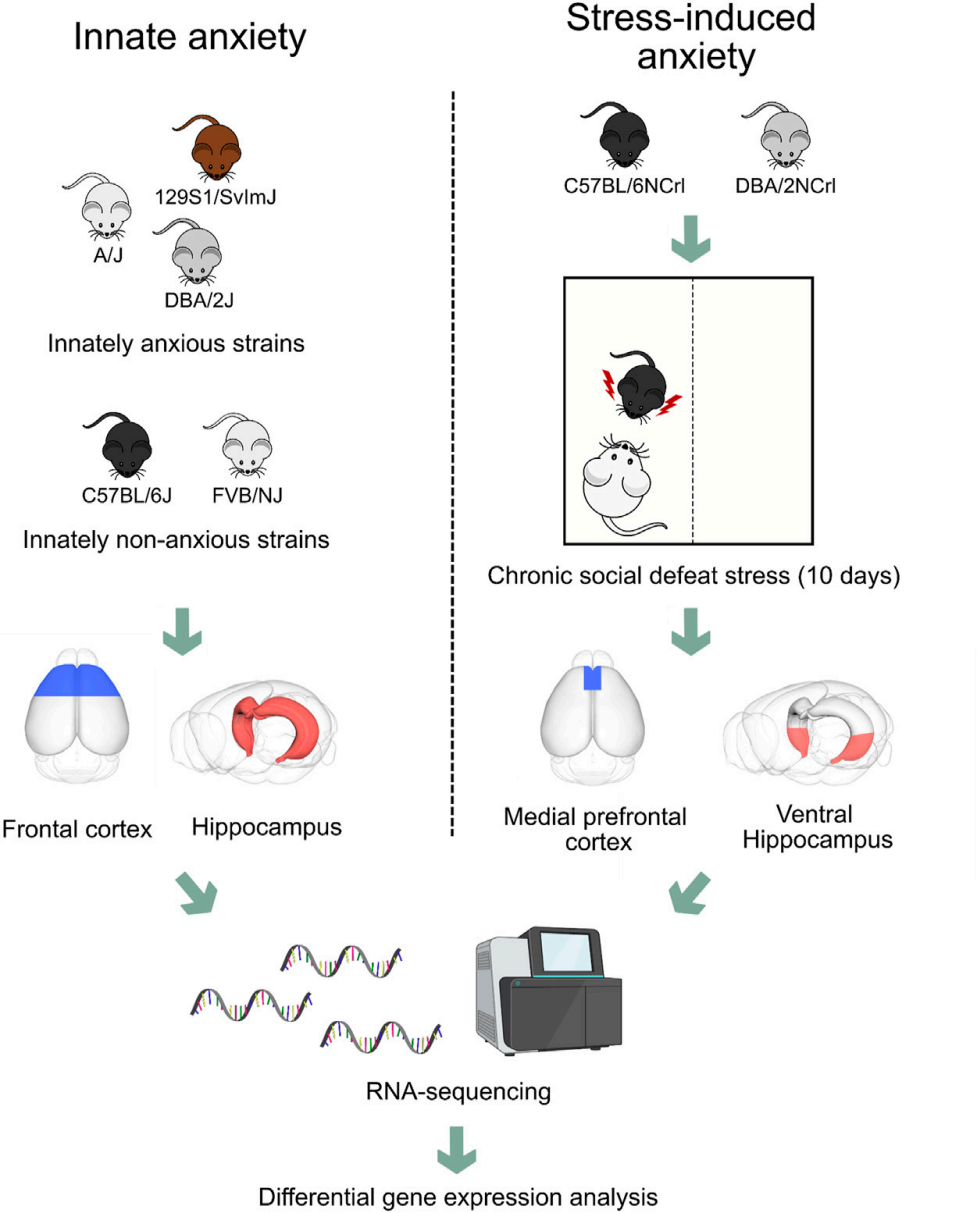
Experimental design to compare gene expression patterns of frontal cortex and hippocampus in innate and stress-induced anxiety. We compared gene expression patterns obtained from the frontal cortex and hippocampus of three innately anxious (A/J, 129S1/SvImJ, and DBA/2J) and two innately non-anxious (C57BL/6J and FVB/NJ) inbred mouse strains with gene expression patterns obtained from the medial prefrontal cortex and ventral hippocampus of C57BL/6NCrl and DBA/2NCrl mice that experienced chronic social defeat stress. Briefly, mice were introduced for 5–10 min into the cage of a resident mouse, which displayed aggressive behavior towards the intruder. The mouse was then moved to a separate part of the cage allowing sensory exposure for 24 h. This stress procedure was repeated for 10 days. On day 11, we performed the social avoidance test to classify the mice as stress-susceptible or resilient based on the extent of their social interaction. In this study, we analysed data from stress-susceptible and non-stressed control mice.

**FIGURE 2:**
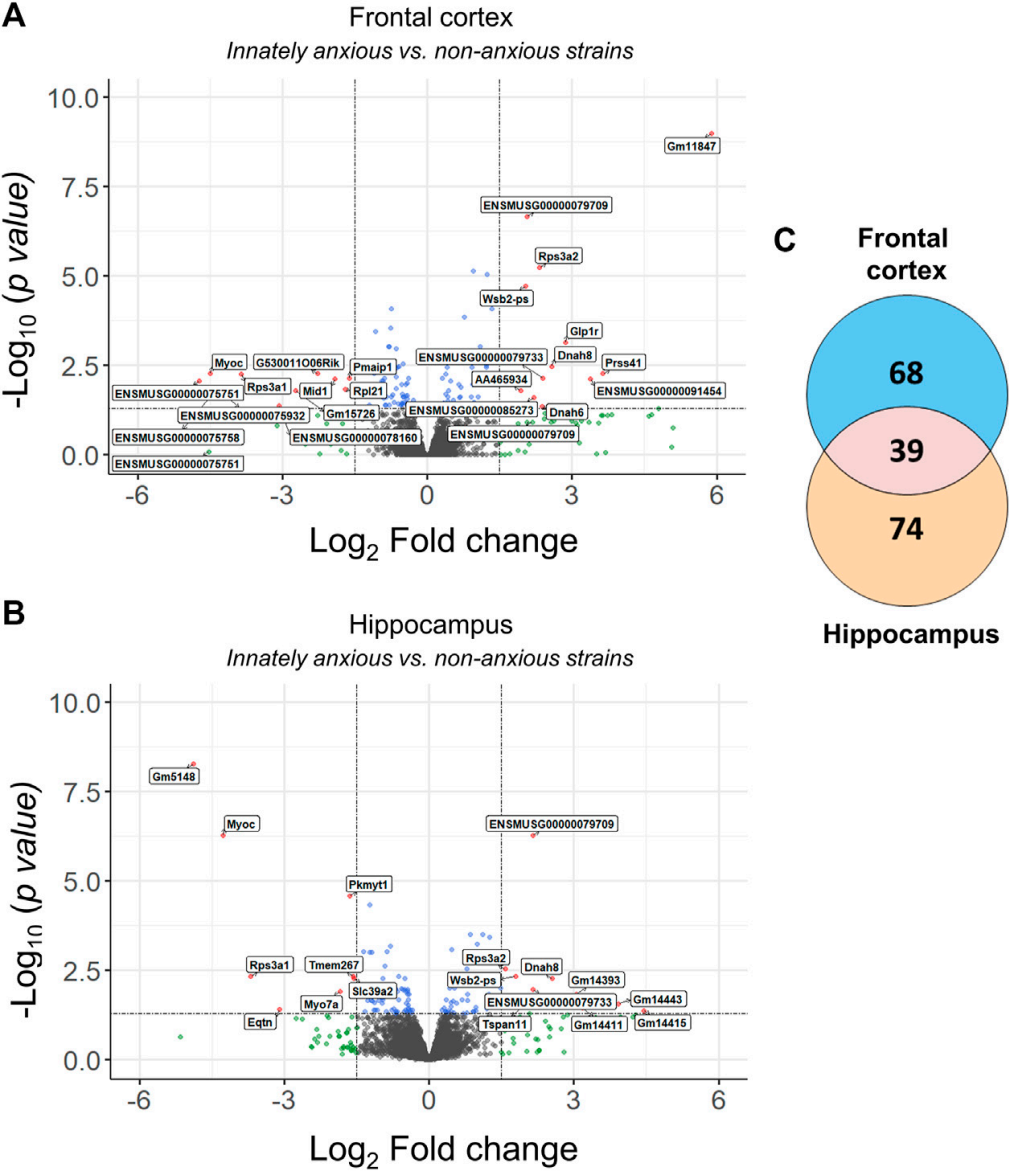
Differentially expressed genes in innately anxious vs. non-anxious inbred mouse strains. **(A–B)** Volcano plots displaying the distribution of differentially expressed genes between the innately anxious vs. non-anxious strains in the FCx **(A)** and Hpc **(B)**. Red = adjusted *p*-value < 0.05 and |logFC| > 1.5, blue = adjusted *p*-value < 0.05 and green = |logFC| > 1.5. **(C)** Venn diagram showing the overlap of differentially expressed genes in the FCx and Hpc (adjusted *p*-value < 0.05). Genes with adjusted *p*-value < 0.05 and |logFC| > 1.5 are labelled with the gene symbol.

We first analysed RNA-sequencing data from the mouse model of innate anxiety-like behavior, consisting of five inbred mouse strains with either high (A/J, 129S1/SvImJ and DBA/2J) or low (C57BL/6J and FVB/NJ) anxiety levels (Figure 1). We have previously determined anxiety-like behavior of these strains using the open field and light dark box tests (Hovatta et al., 2005). To identify differentially expressed genes, we compared samples from the innately anxious strains to the samples from non-anxious strains in the FCx and Hpc. We found 107 and 113 differentially expressed genes in the FCx and Hpc, respectively, with 35% being common to both brain regions (adjusted *p*-value <0.05; Figure 2; Supplementary Table S1).

### 3.2 Mouse model of stress-induced anxiety-like behavior

To identify genes differentially expressed in stress-induced anxiety, we used the CSDS paradigm. It is a 10-day psychosocial stress model based on the confrontation of two male mice, in which an intruder mouse (the test mouse) is placed in the home cage of a resident aggressive CD1 mouse for 5–10 min daily, followed by 24 h of sensory contact. CSDS induces long-term structural and functional changes in the brain (Tsankova et al., 2006; Krishnan et al., 2007). Here, we re-analysed our previously published RNA-sequencing dataset, collected from the medial prefrontal cortex and ventral hippocampus of C57BL/6NCrl and DBA/2NCrl mice that experienced CSDS (Laine et al., 2018) (Figure 1). We focused on the animals that were assigned as stress-susceptible, i.e., that exhibited social avoidance behavior after CSDS, and compared them to non-stressed control mice of the same strain. In the medial prefrontal cortex, 3,468 gene were differentially expressed between C57BL/6NCrl susceptible vs. control mice, and 779 between DBA/2NCrl susceptible vs. control mice (nominal *p*-value <0.05, Supplementary Table S2). In the ventral hippocampus, 10,016 genes were differentially expressed between C57BL/6NCrl susceptible vs. control and 3,597 genes between DBA/2NCrl susceptible vs. control mice (nominal *p*-value < 0.05). The overlap of the number of differentially expressed genes between the medial prefrontal cortex and ventral hippocampus was 18.3% in C57BL/6NCrl susceptible vs. control mice and 3.5% in DBA/2NCrl susceptible vs. control mice.

### 3.3 Gene expression differences between innate and stress-induced anxiety-like behavior

**FIGURE 3:**
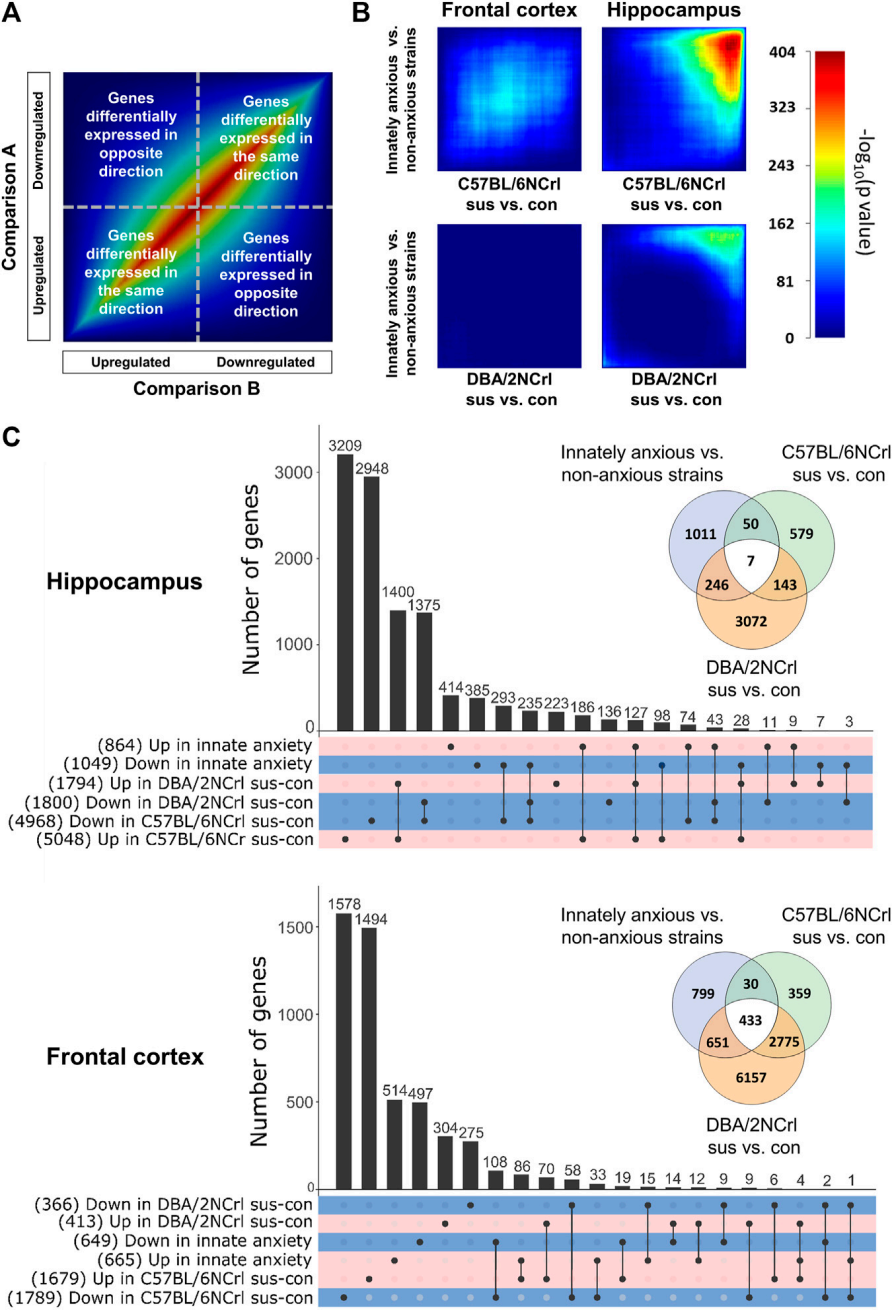
Gene expression overlap of innate and stress-induced anxiety in the frontal cortex and hippocampus. **(A)** Key to Rank Hypergeometric Overlap (RRHO) analysis showing a heatmap of two identical datasets (“Comparison A and Comparison B”). Genes are ranked into bins by their (-log10(*p*-value)*logFC) after differential gene expression analysis and significance of the overlap between different gene bins is compared. **(B)** Results from the RRHO analysis. RRHO shows a unique gene expression signature between innately anxious vs. non-anxious and stress-induced anxiety in the frontal cortex. Highly significant overlap was observed in the hippocampus for the downregulated genes. Scale bar = –log (*p*-value) of rank bins (n = 100). **(C)** UpsetR plot of significantly upregulated (positive logFC) or downregulated (negative logFC) genes (nominal *p*-value < 0.05) in the hippocampus (upper panel) and frontal cortex (lower panel) of innately anxious vs. innately non-anxious strains, C57BL/6NCrl susceptible vs. control, and DBA/2NCrl susceptible vs. control mice. Total number of genes included in each set is shown in parenthesis. Venn diagrams show the extent of the overlap of all differentially expressed genes (nominal *p*-value < 0.05) in innately anxious, non-anxious, and stress-induced anxiety samples.

To investigate the extent of overall gene expression differences in innate and stress-induced anxiety-like behavior we conducted Rank Hypergeometric Overlap (RRHO) analysis. RRHO compares two distinct ranked differential gene expression lists to identify coordinated changes in gene expression in a threshold-free manner (Sb et al., 2010). We carried out this analysis comparing differentially expressed genes in C57BL/6NCrl susceptible vs. control or DBA/2NCrl susceptible vs. control mice to those differentially expressed in innately anxious vs. innately non-anxious mice in both brain regions (Figure 3). We used heatmaps to visualize the significance of the pattern overlap of bins of 100 genes. In general, highly similar gene expression changes in the two compared differential expression gene lists produce a signal across the heatmap’s diagonal (Figure 3A). In the Hpc, but not in the FCx, we detected a significant overlap of genes that were downregulated in both innate and stress-induced anxiety (Figure 3B).

Next, we asked if innate and stress-induced anxiety share highly differentially expressed genes (adjusted *p*-value < 0.05 and |logFC| > 3, Supplementary Tables S1, S2). We did not identify such genes in the FCx, but in the Hpc three genes were differentially expressed both in the innate and stress-induced anxiety: *Myoc* (innate anxiety; logFC = - 4.3, adjusted *p*-value = 5.31E-07, C57BL/6NCrl sus vs. con; logFC = −3.36, adjusted *p*-value = 1.96E-14), *Eqtn* (innate anxiety; logFC = −3.11, adjusted *p*-value = 0.0396, C57BL/6NCrl sus vs. con; logFC = −4.54, adjusted *p*-value = 5.74E-15), and *Gm5148* (innate anxiety; logFC = −4.89, adjusted *p*-value = 5.42E-09, C57BL/6NCrl sus vs. con; logFC = −3.84, adjusted *p*-value = 1.86E-18).

We then examined the degree of overlap of all differentially expressed genes (nominal *p*-value < 0.05) between innate and stress-induced anxiety (Figure 3C). The largest up and downregulated differentially expressed gene sets were exclusive to innate or stress-induced anxiety. Concurring with the RRHO findings, the number of differentially expressed genes shared between innate anxiety and stress-induced anxiety comparisons was higher in the Hpc compared to the FCx (57.6% and 22.5%, respectively, Figure 3C). Taken together, these results indicate that innate and stress-induced anxiety-like behavior display a similar directional expression of genes in the Hpc but largely differ in terms of the FCx gene expression.

### 3.4 Biological pathways associated with innate and stress-induced anxiety-like behavior

**FIGURE 4:**
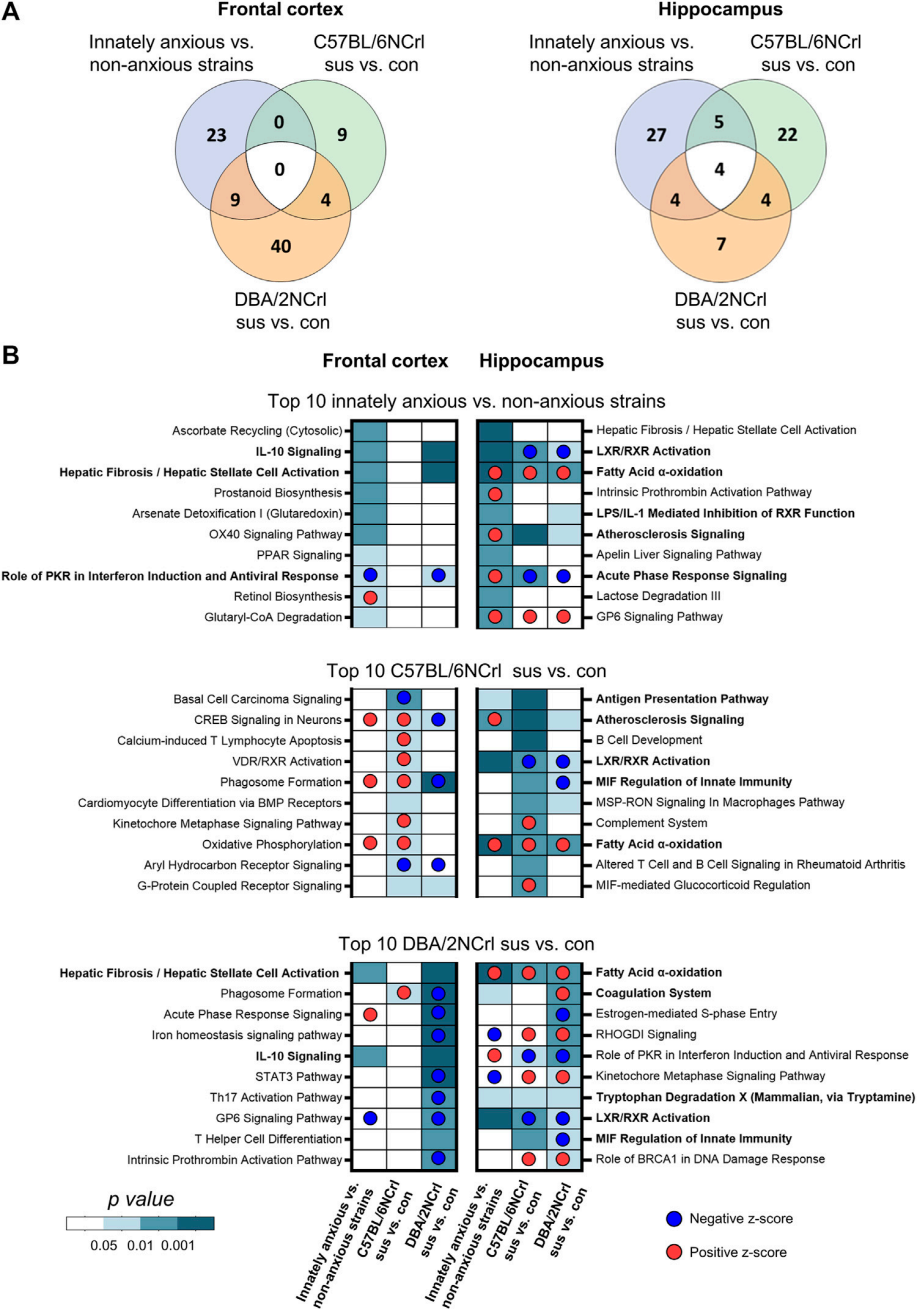
Ingenuity Pathway Analysis (IPA) of enriched biological pathways in innate and stressed-induced anxiety-like behavior converge onto inflammation and immunity. **(A)** Venn diagrams showing the overlap of all significantly enriched canonical pathways (nominal *p*-value < 0.05) between comparisons in the frontal cortex (left diagram) and hippocampus (right diagram). **(B)** Heatmaps showing the 10 most significantly enriched biological pathways (nominal *p*-value) in innately anxious vs. non-anxious, C57BL/6NCrl stress-susceptible vs. control, and DBA/2NCrl stress-susceptible vs. control comparisons in the frontal cortex (left column) and hippocampus (right column). Pathways in bold are significantly enriched in innately anxious vs. non-anxious comparison and in at least one of the stress-induced anxiety comparisons. The most significant 1,000 differentially expressed genes [ranked by their -log10(*p*-value)*logFC] was used as gene list input for IPA.

Having identified that the overall gene expression patterns in innate and stress-induced anxiety are significantly similar in Hpc but differ in the FCx, we next asked which biological pathways the differentially expressed genes are associated with. We carried out pathway analysis using the Ingenuity Pathway Analysis (IPA) to identify enriched biological pathways based on the 1,000 most significantly differentially expressed [-log10 (*p*-value)*logFC] genes. We compared innately anxious vs. non-anxious strains, C57BL/6NCrl stress-susceptible vs. control mice, and DBA/2NCrl stress-susceptible vs. control mice. IPA allows the identification of biological functions or pathways in which the input genes are significantly enriched (*p*-value < 0.05). This is done by comparing the input gene list to the genes known to be associated with biological functions or pathways in the Ingenuity® Knowledge Base. The Knowledge Base consists of curated evidence from the literature and over 30 third party databases, including the Gene Ontology (GO) categories and Kyoto Encyclopedia of Genes and Genomes (KEGG) pathways. In addition, based on the directional changes of the enriched genes, IPA calculates an activation z-score for each biological function, predicting its activity to be increased (z-score > 0) or decreased (z-score < 0) (Krämer et al., 2014). For example, if a set of genes are known to inhibit a pathway, their higher expression levels will lead to the prediction of a decreased biological function. We found that of the significantly enriched pathways, 11.5% in the FCx and 17.1% in the Hpc were shared between innate and stress-induced anxiety-like behavior of either strain (nominal *p*-value < 0.05, Figure 4A). Among the 10 most significantly enriched pathways in innate or stress-induced anxiety, we observed 3 pathways in the FCx and 9 pathways in the Hpc that were common to innate anxiety and at least one of the stress-induced anxiety comparisons (Figure 4B, pathways in bold). Notably, 6 of these 12 pathways were associated with inflammation and immune response, including the “IL-10 signaling”, “LXR/RXR Activation”, “Acute Phase Response Signaling”,“Antigen Presentation Pathway”, “LPS/IL-1 Mediated Inhibition of RXR Function”, and “MIF Regulation of Innate Immunity”. “Fatty Acid α-oxidation” was the only signalling pathway that was common to innate and stress-induced anxiety comparisons in the Hpc.

**FIGURE 5:**
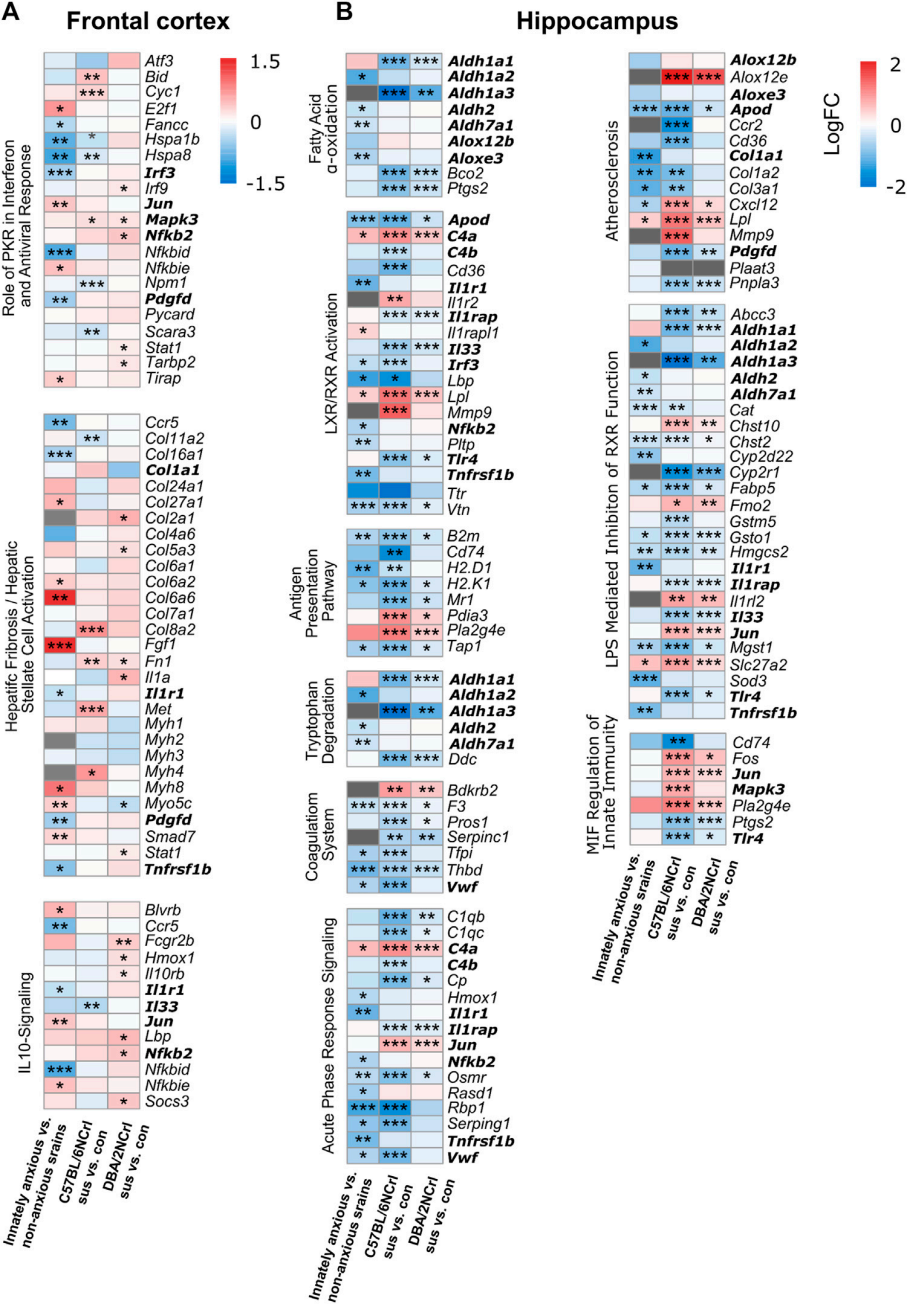
Differential expression levels of genes associated with enriched pathways common for innate and stress-induced anxiety-like behavior. **(A–B)** Heatmaps showing the expression changes (logFC) of genes associated with the statistically enriched biological pathways in innately anxious vs. non-anxious and in at least one of the stress-induced anxiety comparisons (pathways bolded in Figure 4B), in the frontal cortex **(A)** and hippocampus **(B)**. Genes that did not pass the detection threshold in the given data set are marked with gray cells. Genes in bold are found in several pathways. Nominal *p*-value ≤ 0.05 *, < 0.01 **, < 0.001 ***.

We next examined in more detail the 12 pathways that were common to innate and stress-induced anxiety. While the same biological pathways were altered in innate and stress-induced anxiety, individual differentially expressed genes (nominal *p*-value < 0.05) within each pathway may be distinct. Indeed, we found that the majority of the genes, 56% in the FCx and 62% in the Hpc, within these pathways were exclusively differentially expressed in innate or stress-induced anxiety-like behavior, but not both (Figures 5A,B). Altogether, the gene expression differences of innate and stress-induced anxiety-like behavior mostly associated with different canonical pathways. However, gene expression patterns at least partly converged to common pathways associated with inflammation and immunity, suggesting that these biological processes are altered in innate and stress-induced anxiety alike.

### 3.5 Gene-drug interaction analysis of differentially expressed genes in innate and stress-induced anxiety-like behavior

**FIGURE 6:**
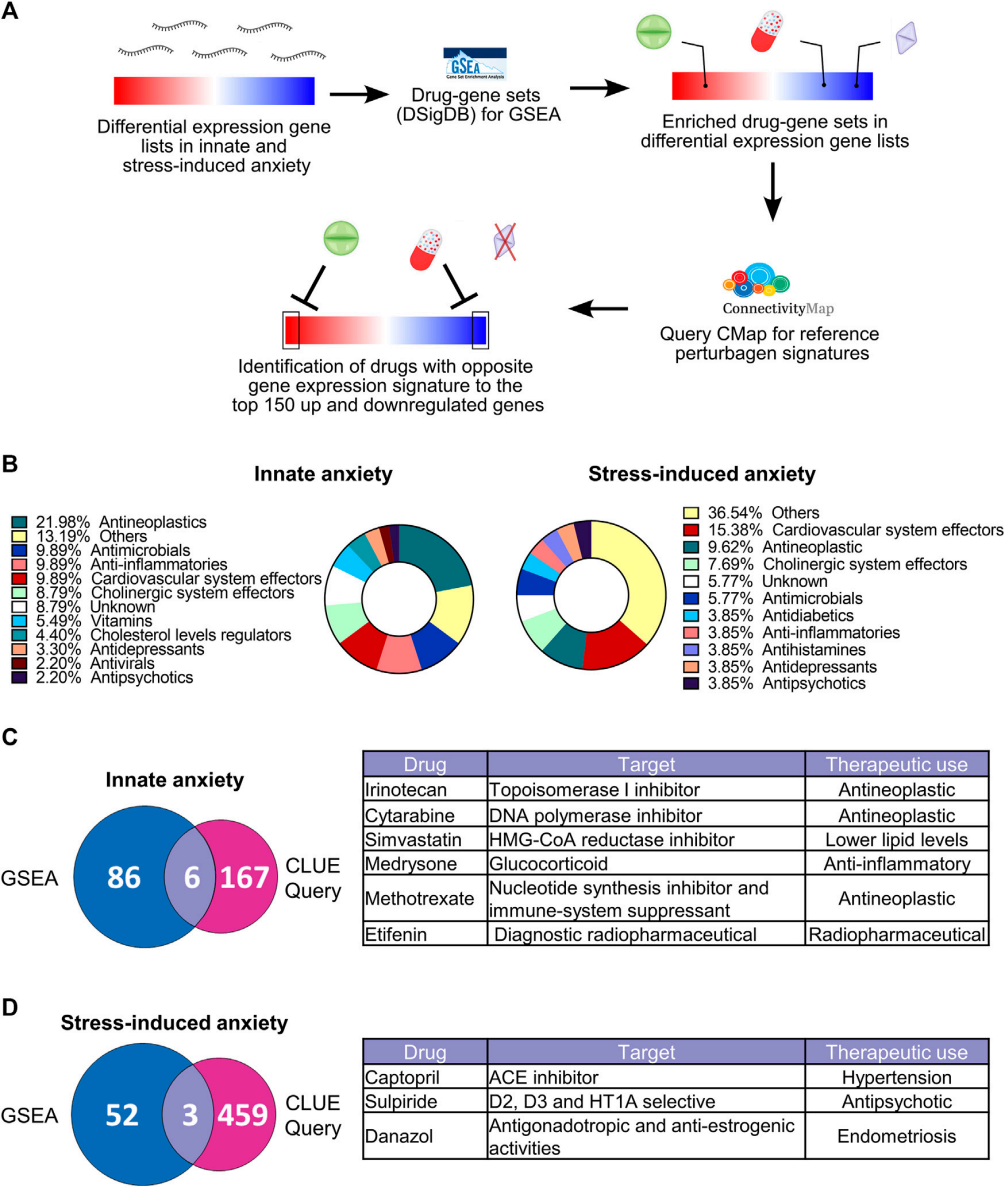
Gene-drug enrichment analysis in innate and stress-induced anxiety-like behavior. **(A)** Gene-drug enrichment was carried out using the Drug Signatures DataBase (DSigDB) gene sets in the Gene set enrichment analysis (GSEA). GSEA allows unbiased identification of drugs with gene expression signatures that are significantly enriched at the top or the bottom of differentially expressed gene lists (familywise-error rate (FWER) < 0.05)). For the drugs identified by GSEA, we next conducted the CLUE Query from the CMap to identify which of these drugs have opposite gene expression signatures to our datasets, using the top 150 up and downregulated genes [ranked by -log10(*p*-value)*logFC)] for each comparison (false-rate discovery < 0.05 and connectivity score < 0). The “Others” drug category contains drugs that were unique to their therapeutic class (Supplementary Table S3). **(B)** Therapeutic categories of drugs significantly enriched [familywise-error rate (FWER) < 0.05] after GSEA based on the gene expression signatures of innate (left) and stress-induced anxiety-like behavior (right). Complete list of drugs is available in Supplementary Table S3. **(C–D)** Overlap of drugs identified after GSEA and CLUE Query analysis based on the gene expression signatures of innate **(C)** and stress-induced **(D)** anxiety-like behavior. Tables show drugs identified in both GSEA and CLUE Query analysis. Drugs identified by GSEA and CLUE Query represent results from both FCx and Hpc.

Gene expression data has been used for drug discovery and drug repurposing (Yang et al., 2020). We reasoned that gene expression signatures associated with innate or stress-induced anxiety-like behavior may be used to identify compounds with potential anxiolytic or anxiogenic effects. We thus investigated whether the gene expression signatures of innate and stress-induced anxiety-like behavior map to drugs and compounds that act on the detected biological pathways. To identify these, we conducted drug-target enrichment analysis by carrying out Gene Set Enrichment Analysis (GSEA) using the Drug Signatures Database (DSigDB) as gene sets database for the GSEA (Figure 6A). DSigDB is a gene expression dataset that depicts changes obtained after exposing multiple cell lines to different drugs and compounds (Yoo et al., 2015). We combined the results of drug-target enrichment analysis for both the FCx and Hpc. Gene expression signatures of innate and stress-induced anxiety were enriched for drugs that belonged to the same therapeutic categories (Figure 6B; Supplementary Table S3). We identified “Vitamins”, “Antivirals”, and “Cholesterol level regulators” therapeutic categories uniquely associated with the gene expression patterns of innate anxiety. “Antidiabetics” were exclusively associated with the gene expression signatures of stress-induced anxiety, suggesting that certain drug categories might be of interest to modulate innate or stress-induced anxiety behaviors.

GSEA analysis does not allow to predict in which direction the significantly enriched drug-gene sets might influence the expression levels of genes in the associated drug-gene sets. In addition, enriched drug-gene sets may contain a large number of genes that are not differentially expressed in our data. To identify the most potent compounds with an anxiolytic effect, we used the top 150 most up and downregulated genes [ranked by -log10 (*p*-value)*logFC] in innate and stress-induced anxiety comparisons as input lists for CLUE Query analysis (CMap) (Subramanian et al., 2017). This cloud-based software allows identification of drug-induced gene expression changes with opposite or similar directionality to those observed in anxiety. CMap contains gene expression profiles from > 5,000 compounds tested in multiple cell lines. For this reason, we restricted our selection for drugs that had both their gene-sets enriched after GSEA and induced significantly opposite gene expression signatures to our differentially expressed gene lists in the CLUE Query analysis (FDR < 0.05 and connectivity score < 0, Figures 6C,D). Fifty percent of the drugs matching these criteria in the innately anxious vs. non-anxious comparison (simvastatin, medrysone, and methotrexate) and all drugs in stress-induced anxiety comparisons target biological processes known to be dysregulated in anxiety disorders, such as immune and inflammatory processes and dopaminergic systems (Borrow and Handa, 2017; Michopoulos et al., 2017; Baik, 2020; Molero et al., 2020; Nagai et al., 2022). Taken together, these results suggest that gene expression signatures of innate and stress-induced anxiety-like behavior associate with different drugs, which, however, have similar modes of action.

## 4 Discussion

In this study, our aim was to explore the similarities and differences in the brain gene expression patterns of innate and stress-induced anxiety-like behavior. We discovered that innate and stress-induced anxiety had, by large, unique fronto-cortical and hippocampal gene expression patterns. This was most evident in the FCx, whereas especially the downregulated genes in the Hpc were the same in innate and stress-induced anxiety-like behavior. In the Hpc, we identified immunological and inflammation processes as the most significantly enriched pathways to converge in innate and stress-induced anxiety. In addition, we found that different individual genes contributed to their enrichment, suggesting that chronic psychosocial stress alters expression of genes that are not influenced by high levels of innate anxiety. We also investigated which drugs or compounds produce similar or opposite gene expression patterns to our data sets, and observed that they were distinct for innate and stress-induced anxiety but affected the same biological processes.

We found large differences in the FCx but significant similarities in the Hpc overall gene expression patterns of innate and stress-induced anxiety-like behavior. The hippocampus is known to encode contextual information, is necessary for the acquisition and retrieval of extinction of fear-associated memories (Maren and Holt, 2000; Bissiere et al., 2011), and coordinates information processing and filtering in the limbic network (Çalışkan and Stork, 2019). While the role of the FCx in stress response is complex, since different anatomic subdivisions play different roles (Sullivan and Gratton, 2002; Radley et al., 2006), it influences emotional, social, and goal-directed behaviors (Cole et al., 2022). In our previous study, using the same dataset of stress-induced anxiety-like behavior (Laine et al., 2018), we found high overlap in differential gene expression of C57BL/6NCrl and DBA/2NCrl susceptible mice after CSDS in the ventral hippocampus but not in the medial prefrontal cortex. In the current study, the hippocampal gene expression patterns were similar between both C57BL/6NCrl and DBA/2NCrl susceptible mice and mice with innate anxiety-like behavior. The Hpc is known to exert both synaptic and neuroendocrine inhibitory feedback on the hypothalamic-pituitary-adrenal (HPA) axis and is a key brain region for the modulation of anxiety (Jacobson and Sapolsky, 1991; Cole et al., 2022).

At this point, we can only speculate that these consistent brain region differences may reflect the different roles of these brain regions in the regulation of anxiety. We found that the overlap of the gene expression patterns of innate and stress-induced anxiety in the Hpc was only partially reflected at the canonical pathway level as 1) pathways enriched in innate and stress-induced anxiety-like behavior were mostly different but 2) converged to inflammation and immune processes as the most significantly enriched pathways. Inflammation and immune processes have been associated with anxiety disorders (Felger, 2018; Wang et al., 2018), including elevated inflammatory markers in the cerebrospinal fluid and circulating concentrations of inflammatory cytokines, chemokines, and acute phase proteins, such as TNF-α, reactant C-reactive protein, and interleukine-6 (Capuron and Miller, 2011; Michopoulos et al., 2017). In our pathway analysis, “Acute Phase Response Signaling” was predicted to be inhibited in innate anxiety but activated in stress-induced anxiety. This signalling pathway is involved in the immediate and non-specific inflammatory response and its activation results from increased production of pro-inflammatory cytokines and acute phase proteins. We observed differential expression of several interleukin receptor genes (e.g., *Il1r1*, *Il1r2*, *Il1rap*) and downstream effectors (*Jun*, *Mapk3*, *Nfkb2*). Our results concur with increased neuroinflammation response in stress-induced anxiety (Won and Kim, 2020) and suggest that this response may be similarly promoted by innate anxiety-like behavior. In mice, systemic pro-inflammatory molecules can trigger a neuroinflammatory response (Breder et al., 1988; Gyoneva et al., 2014). However, the production of cytokines and the kinetics of leukocyte recruitment after an immune challenge is highly dependent on the inbred mouse strain (Hoover-Plow et al., 2008). Thus, the genetic background may modulate the immune system function in both innate and stress-induced anxiety-like behavior.

In the FCx, the gene expression patterns of innate and stress-induced anxiety were mostly dissimilar. This observation was also reflected at the biological pathway level, where the most significantly enriched pathways were non-overlapping. However, these pathways were still associated with neuroinflammation and immune processes. For example, “Prostanoid Biosynthesis” and “OX40 Signaling Pathway” were exclusively enriched in innate anxiety while “Calcium-induced T Lymphocyte Apoptosis” and “VDR/RXR activation” were only enriched in stress-induced anxiety. Thus, innate and stress-induced anxiety may associate with different biological events of the neuroinflammation response. The most enriched biological pathways not associated with neuroinflammation and immune responses encompassed various biological processes. We found “Ascorbate Recycling” to be uniquely significantly enriched in innate anxiety. Lower levels of these vitamins have been found in people suffering from anxiety disorders (Gautam et al., 2012; Mikkelsen et al., 2018; Chang et al., 2019; Silva et al., 2021), and vitamin C supplementation reduces anxiety symptoms (de Oliveira et al., 2015). The biological processes linking vitamin C with anxiety regulation is still poorly understood but could be mediated through its antioxidant properties or modulatory effects on the immune function (de Oliveira et al., 2015). In stress-induced anxiety, non-inflammatory biological pathways were associated with “Hepatic Fibrosis/Hepatic Stellate Cell activation” (containing genes associated with the extracellular matrix), and “Oxidative Phosphorylation”, which have previously been linked to anxiety (Freitag et al., 2003; Hollis et al., 2015; Misiewicz et al., 2019; Blanco and Conant, 2021). Overall, these results demonstrate that highly different gene expression patterns in innate and stress-induced anxiety may converge onto common biological pathways in large. IPA analysis identified the “Fatty acid alpha oxidation pathway” as the only convergent signaling pathway between innate and stress-induced anxiety and it was predicted to be inhibited in all comparisons. In mammals, fatty acid α-oxidation is a lipid metabolism pathway that degrades phytanic acid, a by-product of chlorophyl (Jansen and Wanders, 2006). It occurs pre-dominantly in peroxisomes, which play a central role in generation and detoxification of reactive oxygen species (ROS) (Schrader and Fahimi, 2006; Talley and Mohiuddin, 2022). While peroxisome-related pathways and brain oxidative stress has been linked with anxiety by numerous studies (Hovatta et al., 2005; Bouayed et al., 2009; Locci and Pinna, 2019), the possible direct involvement of fatty acid α-oxidation in anxiety, to our knowledge, has not been studied. Our result supports evidence linking the brain redox status to anxiety-like behavior and propose fatty acid α-oxidation as a convergent biological pathway involved in innate and stress-induced anxiety.

Our gene-drug enrichment analysis revealed that the gene expression signatures of innate, but not stress-induced anxiety-like behavior, resembled gene expression signatures induced by five vitamins (vitamin K precursor and vitamins B1, C, A, and D). In line with our IPA results, we also observed enrichment of genes affected by anti-inflammatory drugs. A meta-analysis of 20 clinical trials (Kappelmann et al., 2018) indicated that anti-cytokine treatment significantly lower anxiety symptoms. However, the molecular mechanisms underlying the effects of vitamin supplements or anti-inflammatory treatment on anxiety symptoms are poorly understood.

We also investigated whether any drugs induce gene expression patterns that are opposite to those of innate or stress-induced anxiety because such drugs may have anxiolytic effects. Gene expression signatures of innate and stress-induced anxiety-like behavior associated with distinct drugs, suggesting that different therapeutic strategies may be considered to alleviate innate and stress-induced anxiety levels. Amongst the drug candidates identified based on the gene expression signatures observed in innate anxiety, captopril has been reported to have anxiolytic effects in both animal models and humans (Costall et al., 1990; Braszko et al., 2003). Sulpiride and danazol might also be of interest, even if reports of their anxiolytic effects are conflicting (Nagai et al., 2022; Bell et al., 2013; Watts et al., 1987). We identified three compounds predicted to produce opposite gene expression changes to stress-induced anxiety: simvastatin, medrysone, and methotrexate. Simvastatin is a cholesterol-lowering statin. Altered lipid and cholesterol levels in the serum have been consistently reported in patients suffering from anxiety disorders. Patients with anxiety disorders have high serum cholesterol levels while patients with major depressive disorders, particularly suicidal patients, have low serum cholesterol levels (Olusi and Fido, 1996; Peter et al., 1999; Papakostas et al., 2004). While several statins have been reported to have adverse side effects on anxiety and depression symptoms, others, such as simvastatin, are proposed to be protective (Molero et al., 2020; De Giorgi et al., 2022), suggesting additional unknown effects of statins on anxiety and mood regulation. One such possible mechanism could involve the reduction of specific ligand binding and G-protein coupling to serotonin 1A receptors upon statin treatment (Shrivastava et al., 2010). The other identified drugs, medrysone and methotrexate, are potent immune system suppressors and anti-inflammatory drugs, but their potential anxiolytic effects have not been investigated. Additional studies should validate that these drugs produce opposite gene expression signatures to those found here, and address the possible side effect of these drugs that may hinder their use as clinical anxiolytics.

Our study has some limitations. Anxiety-like behavior is regulated by a distributed network of brain regions, yet here we concentrated on only two key brain regions. The anatomical regions investigated in innate anxiety-like behavior were larger (frontal part of the cortex and the entire hippocampus) than those investigated in stress-induced anxiety-like behavior (medial prefontal cortex and ventral hippocampus). This difference might influence the observed gene expression patterns that are an average of all cell types within the dissected area. Since our data derive from bulk RNA-sequencing, we were unable to investigate possible cell-type specific gene expression differences. Also, we analyzed one model of innate anxiety and stress-induced anxiety, and therefore, our results may not generalize to all anxiety models. Another possible limitation relates to the drug-enrichment analysis and the employment of the DSigDB and Connectivity Map databases. In these databases, a significant proportion of the drug-induced gene expression signatures were obtained from cancer cell lines, thus possibly explaining the over-representation of “Antineoplastics” in our findings. To our knowledge, drugs and small molecule-related gene sets do not exist for naïve brain cells. However, our results identified drug therapeutic categories and compounds that have been previously linked to anxiety.

In conclusion, our study identified mostly different gene expression changes between innate and stress-induced anxiety-like behavior in the frontal cortex. Nevertheless, in the hippocampus, our results for innate and stress-induced anxiety-like behavior converged to inflammation and immune pathways, suggesting a brain region-specific effect. Therefore, future efforts should investigate brain region-specific immune responses and neuroinflammation processes and their relation to systemic immune responses and behavior. Identifying the molecular mechanisms bridging immune system-associated pathways to anxiety-like behavior will be critical to the development of novel therapeutic treatments for anxiety disorders.

## Data Availability

The datasets presented in this study can be found in Gene Expression Omnibus (GEO; innate anxiety dataset accession GSE189744 and stress-induced anxiety dataset accession GSE109315).
