# Supplementary material for: Gene expression profiling reveals a role of immune system and inflammation in innate and stress-induced anxiety-like behavior
Source: Front Genet. 2023 May 16;14:1173376. doi: 10.3389/fgene.2023.1173376 (PMC10229056; doi:10.3389/fgene.2023.1173376)
Supplement: Supplementary file 4 [file Image1.pdf]

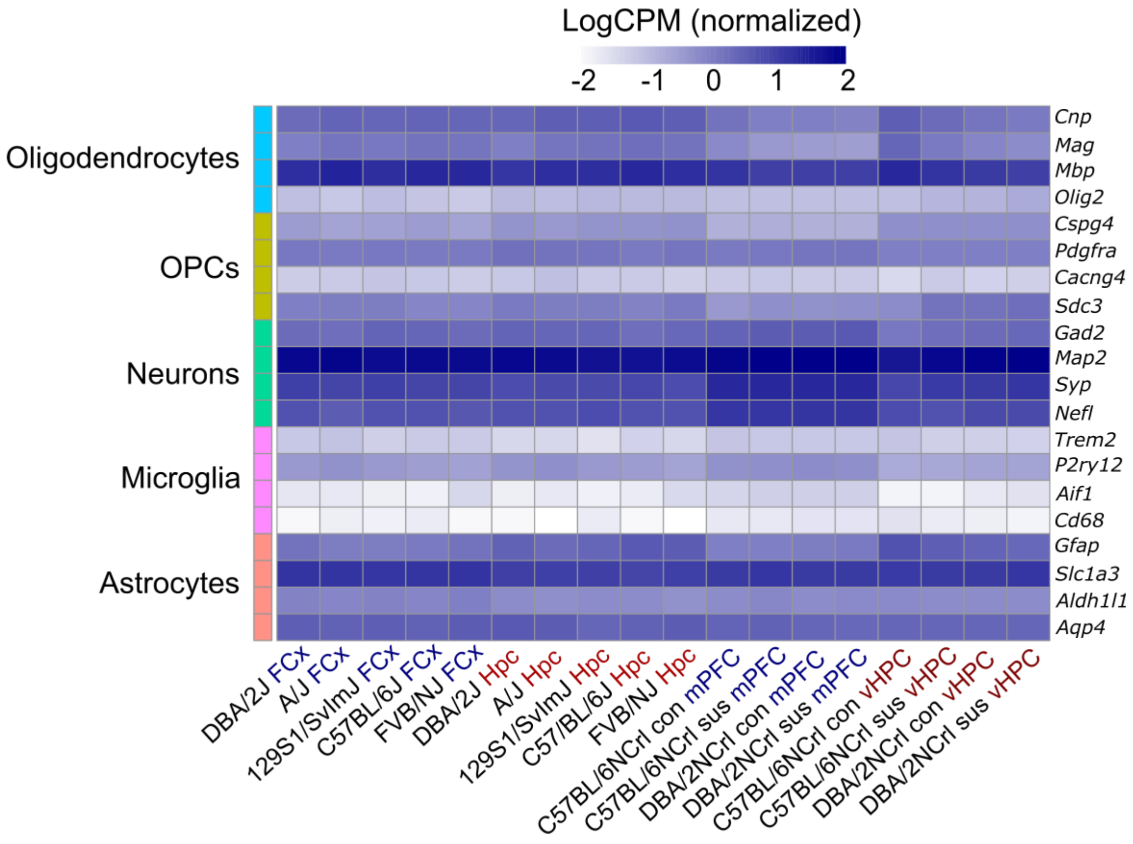

Supplementary Figure 1: Expression levels of brain cell marker genes in the innate and stress-induced anxiety datasets. Heatmap showing the expression levels (voom-normalized logCPM) of known marker genes for oligodendrocytes, oligodendrocyte progenitor cells (OPCs), neurons, microglia, and astrocytes. Frontal cortex (FCx) and hippocampus (Hpc) were analyzed from innately anxious and non-anxious mouse strains. Medial prefrontal cortex (mPFC) and ventral hippocampus (vHPC) were analyzed from mice susceptible (sus) for chronic social defeat stress and unstressed controls (con).
